# Parent-Reported Symptoms and Medications Used Among Children With Severe Neurological Impairment

**DOI:** 10.1001/jamanetworkopen.2020.29082

**Published:** 2020-12-11

**Authors:** James A. Feinstein, Chris Feudtner, Allison B. Blackmer, Robert J. Valuck, Diane L. Fairclough, Jacqueline Holstein, LiseAnne Gregoire, Sadaf Samay, Allison Kempe

**Affiliations:** 1 Adult and Child Consortium for Health Outcomes Research and Delivery Science, University of Colorado and Children’s Hospital Colorado, Aurora; 2 Department of Pediatrics, University of Colorado School of Medicine, Aurora; 3 Division of General Pediatrics, Department of Pediatrics, The Children’s Hospital of Philadelphia, Philadelphia, Pennsylvania; 4 Skaggs School of Pharmacy and Pharmaceutical Sciences, University of Colorado, Aurora; 5 Colorado School of Public Health, Aurora; 6 Research Institute, Children’s Hospital Colorado, Aurora; 7 Research Informatics, Analytics Resource Center, Children’s Hospital Colorado, Aurora

## Abstract

**Importance:**

Children with severe neurological impairment (SNI) often take multiple medications to treat problematic symptoms. However, for children who cannot self-report symptoms, no system exists to assess multiple symptoms and their association with medication use.

**Objectives:**

To assess the prevalence of 28 distinct symptoms, test whether higher global symptom scores (GSS) were associated with use of more medications, and assess the associations between specific symptoms and medications.

**Design, Setting, and Participants:**

This cross-sectional study was conducted between April 1, 2019, and December 31, 2019, using structured parent-reported symptom data paired with clinical and pharmacy data, at a single-center, large, hospital-based special health care needs clinic. Participants included children aged 1 to 18 years with SNI and 5 or more prescribed medications. Data analysis was performed from April to June 2020.

**Exposure:**

During routine clinical visits, parent-reported symptoms were collected using the validated 28-symptom Memorial Symptom Assessment Scale (MSAS) and merged with clinical and pharmacy data.

**Main Outcomes and Measures:**

Symptom prevalence, counts, and GSS (scored 0-100, with 100 being the worst) were calculated, and the association of GSS with medications was examined. To evaluate associations between symptom-medication pairs, the proportion of patients with a symptom who used a medication class or specific medication was calculated.

**Results:**

Of 100 patients, 55.0% were boys, the median (interquartile range [IQR]) age was 9 (5-12) years, 62.0% had 3 or more complex chronic conditions, 76.0% took 10 or more medications, and none were able to complete the MSAS themselves. Parents reported a median (IQR) of 7 (4-10) concurrent active symptoms. The median (IQR) GSS was 12.1 (5.4-20.8) (range, 0.0-41.2) and the GSS was 9.8 points (95% CI, 5.5-14.1 points) higher for those with worse recent health than usual. Irritability (65.0%), insomnia (55.0%), and pain (54.0%) were the most prevalent symptoms. Each 10-point GSS increase was associated with 12% (95% CI, 4%-19%) higher medication counts, adjusted for age and complex chronic condition count. Among the 54.0% of children with reported pain, 61.0% were prescribed an analgesic.

**Conclusions and Relevance:**

These findings suggest that children with SNI reportedly experience substantial symptom burdens and that higher symptom scores are associated with increased medication use. Paired symptom-medication data may help clinicians identify targets for personalized symptom management, including underrecognized or undertreated symptoms.

## Key Points

**Question:**

In children with severe neurological impairment (SNI) who cannot self-report, can comprehensive parent-reported symptom assessments inform medication use?

**Findings:**

In this cross-sectional study of 100 children with SNI and polypharmacy, parents reported that children experienced multiple concurrent high-distress symptoms, notably irritability (65.0%), insomnia (55.0%), and pain (54.0%). Although higher symptom burdens were associated with increasing polypharmacy, opportunities existed to optimize pharmacotherapy; for example, among 54.0% of children with pain, only 61.0% were prescribed an analgesic.

**Meaning:**

Comprehensive parent-reported symptom data paired with medication data could help clinicians identify targets for personalized symptom management, including underrecognized or undertreated symptoms.

## Teaser

This cross-sectional study examines whether higher global symptom scores are associated with use of more medications and assesses associations between specific symptoms and medications among children with severe neurological impairment.

## Introduction

Children with severe neurological impairment (SNI) comprise a high-risk and complex population that has frequent contact with the medical system.^1,2^ Children with SNI have central nervous system diagnoses, such as intractable seizures, cerebral palsy, hydrocephalus, or neurodegenerative processes.^1^ To help these children thrive, clinicians often prescribe multiple medications to treat a variety of problematic and interrelated symptoms, including pain, uncontrolled muscle tone, seizure, sympathetic storming, dyspnea, gastroesophageal reflux, and constipation.^3,4,5,6,7,8,9,10^ Simultaneously, polypharmacy is associated with major morbidity and cost; however, our ability to recognize, monitor, and manage both desired and adverse symptoms associated with specific medications in the context of polypharmacy is severely underdeveloped.^8,11,12,13^ Children with SNI frequently have substantial communication impairment, further limiting clinical assessment of symptoms and evaluation of responses to therapies.^14,15,16^ Accordingly, the Best Pharmaceuticals for Children Act prioritized the need to study outcome measures of medication safety and efficacy in children with intellectual and developmental disabilities.^17,18^

Patient-reported outcomes have increasingly been incorporated into pediatric clinical care and research activities, and these type of data present an opportunity to supplement typical clinical data with more comprehensive, structured symptom data.^19^ Patient-reported and proxy-reported symptom inventories, such as the validated 28-item Memorial Symptom Assessment Scale (MSAS), have measured symptom burdens and health-related quality of life in pediatric oncology and palliative care patients but have not been widely used in other complex pediatric populations.^14,16,20,21,22^ For nonverbal children with SNI who cannot self-report, parents and direct caregivers may be best positioned to complete the proxy MSAS instrument to assess their children’s current symptoms.^19,23,24,25^ Such information could help clinicians to understand the symptoms typically encountered by their patients and, ultimately, optimize their prescribing and monitoring decisions.^16,26^

Thus, in a population of children with SNI and polypharmacy, we analyzed cross-sectional parent-reported symptom data paired with clinical and medication data to provide estimates of the most prevalent parent-reported symptoms among children with SNI, stratified by children’s overall recent health status; tested the hypothesis that greater overall parent-reported symptom burdens are associated with higher levels of polypharmacy; and assessed whether the presence of specific symptoms are associated with use of certain medications. Specifically, we explored 2 important goals of pharmacotherapy: first, the goal of treating symptoms, measured by whether participants with a reported symptom (eg, pain or constipation) used a medication known to treat that symptom (eg, analgesic or laxative); and, second, the goal of avoiding potential adverse symptoms, measured by whether those with a potential adverse symptom (eg, constipation or drowsiness) used a medication known to provoke that symptom (eg, anticholinergics, which are known to cause substantial adverse symptoms in complex adult patients with polypharmacy).^27^

## Methods

### Study Design

This is a cross-sectional study of parent-reported symptoms and medications used in children with SNI and polypharmacy. This study follows the Strengthening the Reporting of Observational Studies in Epidemiology (STROBE) reporting guideline for observational cross-sectional studies.^28^ This study was approved by the Colorado Multiple Institutional Review Board and registered with ClinicalTrials.gov (NCT03849066).

### Identification, Consent, and Enrollment of Participants

Between April 1, 2019, and December 31, 2019, we obtained written, informed parental consent to enroll English- and Spanish-speaking parents and their children aged 1 to 18 years with SNI and polypharmacy who received primary care in a large, hospital-based, special health care needs clinic. Children with SNI have neurological diagnoses that are expected to last 12 months or longer and result in systemic or multisystem physiologic impairment requiring pediatric subspecialty care.^1^ The presence of SNI and complex chronic conditions (CCCs) were identified using published classification systems based on *International Statistical Classification of Diseases, Tenth Revision, Clinical Modification* diagnosis codes.^1,29^ Polypharmacy was defined as using 5 or more scheduled outpatient prescription medications, as detailed later in the article.^30^

### Parent-Reported Symptom Assessments

We implemented a comprehensive data capture system, the Parent-Reported Outcomes of Symptoms (PRO-Sx) system,^31^ to automatically extract and process structured symptom, clinical, and medication data (eFigure 1 in the Supplement). Before the start of a nonacute, routine medical visit, parents reported symptom data regarding their child using a tablet-based implementation of the pediatric-specific proxy-MSAS instrument.^16,21^ For 28 distinct symptoms, parents were asked to report whether their child experienced each symptom in the past 7 days and, if so, the frequency, severity, and perceived extent of bother.^16,21^ Parents also reported their child’s overall health status in the past week (dichotomized to worse state of health vs usual state of health or better) to verify that reported symptoms occurred during usual health and did not represent acutely worsened symptoms. All study data, including parent demographic data and parent-reported race/ethnicity, were collected and managed using REDCap electronic data capture tools hosted at the University of Colorado.^32^

### Clinical and Health Care Utilization Variables

Additional analytic variables were automatically merged from the patient’s electronic health record to the PRO-Sx database after the clinic visit record was closed by the clinician. Variables included patient demographic information, *International Statistical Classification of Diseases, Tenth Revision, Clinical Modification* diagnoses from the visit and from the prior year, previsit reconciled prescription medications, and health care utilization in the prior year (outpatient primary care and subspecialty, emergency, and inpatient medical visit counts).

### Medication Counts and Classifications

Medication counts were calculated on the day of the study visit after medication reconciliation was performed but before the start of the clinical visit.^30^ Consistent with previous studies,^3^ to estimate the number of systemic medications to which a patient was routinely exposed, we excluded as-needed medications, intermittent medications (eg, botulinum toxin injections), inpatient-administered medications (eg, intravenous bisphosphonates), vaccines, and topical, eye, ear, and nose formulations. Medications were classified using the World Health Organization’s Anatomic Therapeutic Chemical classification system.^33^ Because anticholinergic medications span multiple Anatomic Therapeutic Chemical classes, anticholinergics with a pediatric indication were identified from published literature by a pediatric special needs pharmacist (A.B.B.).^27^

### Primary Outcomes

All analyses of MSAS symptom data were performed using conventional scoring methods.^14,16,22,23,34^ Individual symptom scores were calculated by averaging each symptom’s scores for frequency (on a scale of 1 to 4, with higher scores indicating worse status), severity (1 to 4, with higher scores indicating greater severity), and extent of bother (1 to 5, with higher scores indicating more bother) and then standardizing to a 0 to 100 scale.^16^ The main outcome, global symptom score (GSS), was the mean of the 28 individual symptoms (0-100), with higher scores indicating an increased symptom burden.^16^ For analyses of association between GSS and polypharmacy, the primary outcome was the count of prescription medications. Finally, for analyses of associations between specific symptoms and certain types of medications, the primary outcome was use of a medication class (eg, any analgesic medication) or use of a specific medication (eg, oxycodone).

### Statistical Analysis

Descriptive statistics and distributional graphs were used to examine total counts of symptoms, GSS, and individual symptom prevalence. To test whether GSS was associated with a child’s recent health status, we compared group means using 2-sample *t* tests. To test whether GSS was associated with higher levels of polypharmacy, we performed multivariable zero-truncated negative binomial regression with the number of medications as the continuous outcome variable, adjusting for participant age and number of CCCs. Zero-truncated negative binomial regression was used because the count outcome was left truncated at 5 medications (based on study eligibility criteria) and displayed evidence of overdispersion.^35^ We calculated proportions of children with and without a symptom who used specific medications, stratified by recent health, and we tested comparisons using 2-sample tests of proportions. Analyses were conducted using Stata statistical software version 16.1 (StataCorp), and significance was set at a 2-tailed *P* < .05. Data analysis was performed from April to June 2020.

## Results

### Demographic and Clinical Characteristics of Study Population

**Figure 1.**
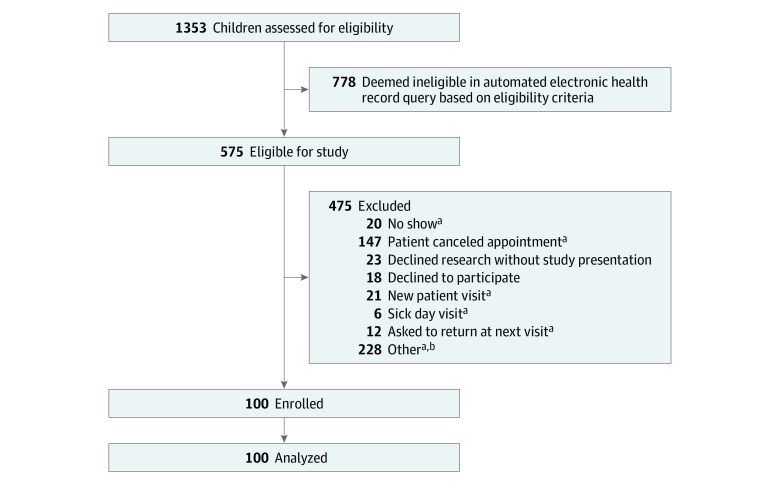
Screening, Eligibility, and Study Inclusion Flowchart ^a^Patient was eligible to be reapproached. ^b^Includes medical team entered immediately, no research assistant available.

One hundred patients with SNI and their parents were enrolled and completed the study visit (Figure 1). Overall, participants had substantial medical complexity (Table 1). Most patients were boys (55.0%), and patients had a median (interquartile range [IQR]) age of 9 (5-12) years and a high prevalence of 3 or more CCCs (62.0%). Health care utilization was high, with prevalent use of 10 or more daily prescription medications (76.0%) and frequent outpatient medical visits (46.0% of patients with ≥20 visits annually), emergency visits (46.0% of patients with ≥2 visits annually), and inpatient stays (50.0% of patients with ≥2 stays annually). No patients were able to assent and complete the symptom inventory themselves. Parents who reported patients’ symptoms were primarily female (83.0%), aged 31 to 40 years (47.0%), were White (78.0%), had a biological relationship to the child (88.0%), spoke English (95.0%), and had some college education (79.0%) (eTable in the Supplement). Most parents (89.0%) provided 7 days per week of direct care.

**Table 1.** Demographic and Clinical Characteristics of Children With Severe Neurological Impairment by Symptom Count and Global Symptom Score

Characteristic	Patients, % (N = 100)	Median (IQR)	
		Symptom count	Global symptom score^a^
Age, y			
1-4	22.0	3 (1-7)	5.0 (1.3-12.5)
5-8	28.0	7 (5-10)	14.4 (7.0-21.1)
9-12	28.0	6 (4-9)	9.7 (5.2-16.5)
13-18	22.0	10 (6-14)	19.9 (10.1-27.1)
Sex			
Male	55.0	6 (3-9)	10.4 (4.8-18.2)
Female	45.0	7 (4-11)	12.9 (5.7-22.8)
Race			
American Indian or Alaska Native^b^	1.0	NA	NA
Black or African American	5.0	4 (3-6)	5.4 (5.1-9.5)
White	75.0	7 (4-11)	12.2 (6.3-22.6)
>1 Race	13.0	8 (4-9)	13.7 (6.3-18.8)
Not specified	6.0	3 (1-5)	4.4 (1.8-8.6)
Ethnicity			
Not Hispanic or Latino	75.0	7 (4-11)	12.5 (5.7-22.6)
Hispanic or Latino	25.0	6 (3-8)	9.8 (4.8-17.9)
Complex chronic conditions, No.^c^			
0	4.0	9 (9-10)	15.8 (14.4-19.1)
1-2	25.0	5 (3-8)	9.2 (5.1-21.1)
3-4	35.0	8 (4-11)	14.0 (5.8-22.8)
≥5	36.0	6 (4-9)	10.3 (5.1-18.8)
Outpatient visits in prior year, No.			
1-9	24.0	6 (3-8)	9.2 (2.7-15.9)
10-19	30.0	6 (2-10)	9.4 (3.9-21.1)
20-29	24.0	8 (4-11)	12.3 (6.0-19.3)
≥30	22.0	9 (6-13)	17.1 (9.8-26.8)
Emergency visits in prior year, No.			
0	39.0	7 (4-9)	12.2 (5.4-18.5)
1	15.0	4 (1-6)	5.7 (0.9-11.0)
2	21.0	6 (4-10)	12.2 (5.8-18.2)
≥3	25.0	10 (5-12)	19.0 (7.7-24.1)
Inpatient stays in prior year, No.			
0	25.0	4 (1-9)	6.3 (0.9-21.1)
1	25.0	6 (4-9)	9.8 (5.4-17.3)
2	18.0	8 (5-11)	14.1 (8.5-21.1)
≥3	32.0	7 (4-12)	12.7 (6.5-24.5)
Medication count before visit			
5-9	24.0	5 (1-9)	8.9 (1.8-15.2)
10-14	39.0	7 (4-9)	12.5 (5.4-18.2)
≥15	37.0	8 (5-12)	16.1 (6.3-23.2)

Abbreviations: IQR, interquartile range; NA, not applicable.

^a^ The global symptom score is scored from 0 to 100, with 100 being the worst.

^b^ Individual scores were not reported because the sample size was less than 5.

^c^ Determined from historical *International Statistical Classification of Diseases, Tenth Revision, Clinical Modification* codes obtained from the electronic health record; severe neurological impairment and complex chronic condition classification schemes are independent systems and a small subset of severe neurological impairment *International Statistical Classification of Diseases, Tenth Revision, Clinical Modification* codes are not contained in the complex chronic condition classification system.

### Symptom Burdens Among Children

Parents reported that in the week before the visit, 77.0% of patients were in their usual state of health or better, whereas 23.0% were worse than usual. Overall, parents reported a median (IQR) of 7 (4-10) concurrent active symptoms (range, 0-18 symptoms) (Table 1). The median (IQR) GSS was 12.1 (5.4-20.8) (range, 0.0-41.2). On average, the unadjusted GSS for a child reported to be in a worse state of health than usual was 9.8 points (95% CI, 5.5-14.1 points) higher compared with the GSS for a child in a usual state of health or better. Overall, the most prevalent individual symptoms were irritability (65.0%), insomnia (55.0%), and pain (54.0%) (Table 2). The highest individual symptom scores were noted for dysphagia (median [IQR], 66.7 [41.7-87.5]), dry mouth (median [IQR], 66.7 [41.7-75.0]), and diarrhea (median [IQR], 62.5 [41.7-75.0]) (Table 2). For all symptoms except skin issues, anorexia, and bleeding, most children with that symptom were in their usual state of health.

**Table 2.** Prevalence of Individual Symptoms by Overall Recent Health Status in Children With Severe Neurological Impairment

Symptom	Prevalence, % (N = 100)	Individual symptom score, median (IQR)^a^	Usual health in prior 7 d, row %
Irritability	65.0	58.3 (50.0-66.7)	72.3
Insomnia	55.0	58.3 (50.0-75.0)	63.6
Pain	54.0	54.2 (50.0-66.7)	66.7
Fatigue	46.0	58.3 (41.7-66.7)	67.4
Cough	41.0	41.7 (33.3-50.0)	63.4
Drowsiness	35.0	50.0 (37.5-58.3)	71.4
Dyspnea	33.0	50.0 (33.3-50.0)	57.6
Constipation	28.0	50.0 (37.5-62.5)	71.4
Nausea	26.0	50.0 (41.7-75.0)	73.1
Concentration	25.0	58.3 (41.7-75.0)	64.0
Nervousness	24.0	54.2 (41.7-75.0)	70.8
Diarrhea	24.0	62.5 (41.7-75.0)	66.7
Vomiting	23.0	50.0 (37.5-75.0)	78.3
Headache	23.0	58.3 (41.7-66.7)	60.9
Sadness	22.0	41.7 (33.3-50.0)	59.1
Seizure	22.0	50.0 (43.8-100.0)	63.6
Worrying	19.0	58.3 (41.7-66.7)	63.2
Itching	19.0	50.0 (41.7-58.3)	78.9
Dysphagia	18.0	66.7 (41.7-87.5)	55.6
Anorexia	17.0	50.0 (41.7-58.3)	41.2
Sweating	16.0	58.3 (35.4-66.7)	75.0
Dry mouth	15.0	66.7 (41.7-75.0)	80.0
Numbness	12.0	45.8 (37.5-58.3)	58.3
Skin issues	11.0	25.0 (25.0-37.5)	45.5
Dysuria	10.0	50.0 (50.0-62.5)	60.0
Bleeding	8.0	50.0 (25.0-75.0)	25.0
Mouth sores	5.0	25.0 (25.0-50.0)	60.0
Image issues^b^	1.0	NA	NA

Abbreviations: IQR, interquartile range; NA, not applicable.

^a^ The individual symptom score is calculated for positive symptoms as the average of the reported symptom frequency, severity, and extent of bother for each symptom. Response options were scored on a 0 to 100 scale (with 100 indicating the worst score).

^b^ Individual scores were not reported because the sample size was less than 5.

### Association of GSS With Polypharmacy

**Figure 2.**
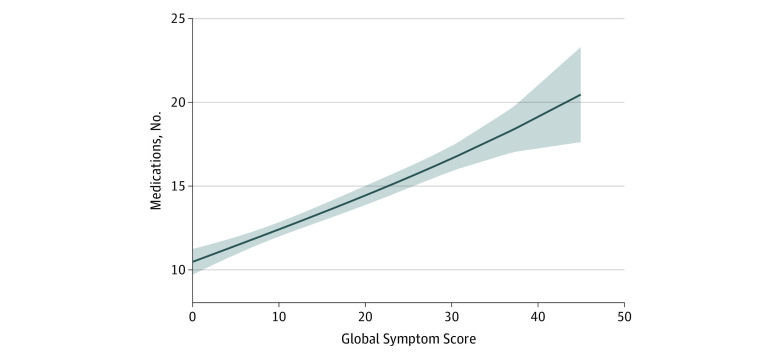
Association Between Global Symptom Score (GSS) and Number of Daily Prescription Medications in 100 Children With Severe Neurological Impairment This graph displays the association between GSS (independent variable) and number of daily prescription medications (dependent variable), adjusted for patient age and number of complex chronic conditions. The shaded area represents the 95% CI. On average, each 10-point increase in GSS was associated with a 12% (95% CI, 4%-19%) increase in total medication counts. The GSS is scored from 0 to 100, with 100 being the worst.

Increasing GSS was associated with increasing polypharmacy, after adjustment for participant age and number of CCCs (Figure 2). On average, each 10-point increase in GSS was associated with a 12% (95% CI, 4%-19%) increase in medication counts. Most patients’ medication regimens included 1 or more neurological, respiratory, and gastrointestinal medication (eFigure 2 in the Supplement).

### Specific Symptoms and Associated Medications

**Figure 3.**
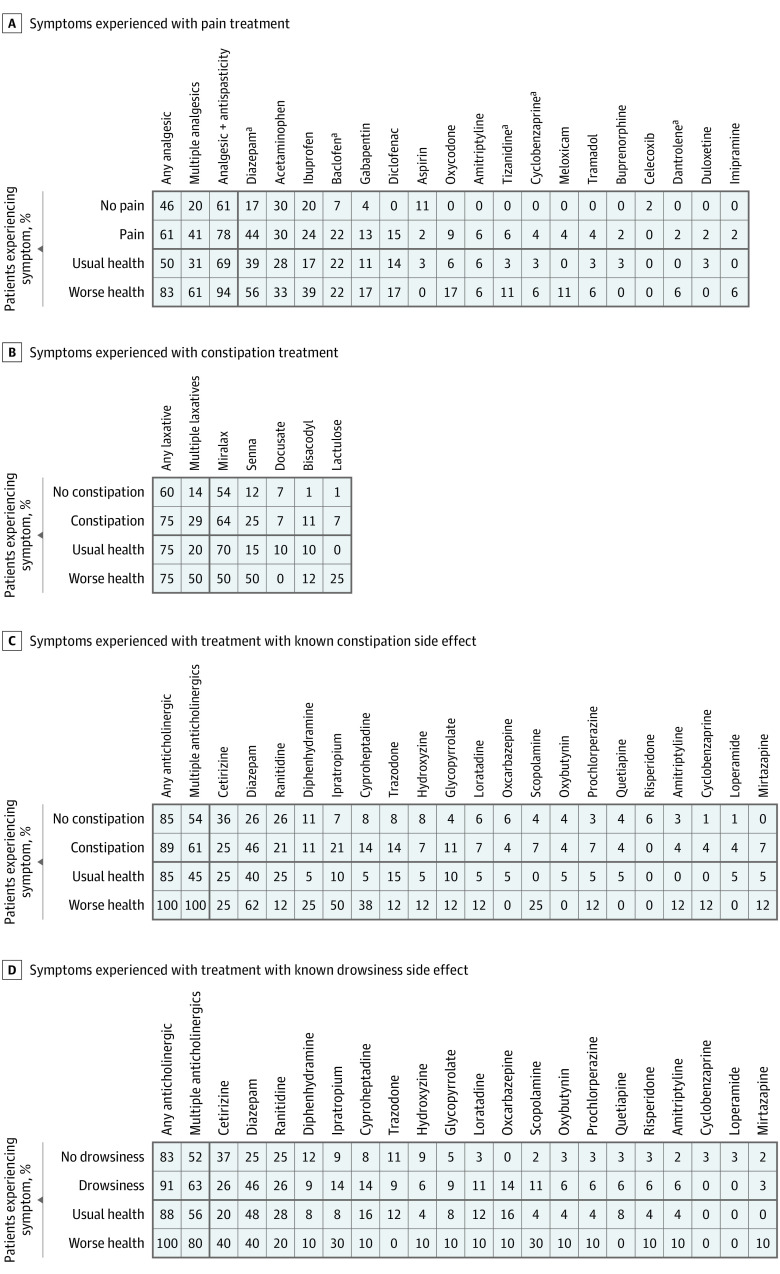
Specific Symptoms Associated with Prescribed Medication Classes in 100 Children With Severe Neurological Impairment The percentage displayed in each square corresponds to the percentage of children with a reported symptom (y-axis) who used a specific medication (x-axis, ordered by descending overall medication utilization). Because of no use, the following medications were not displayed in panel A: naproxen, morphine, methadone, and pregabalin. Similarly, the following medications were not displayed in panels C and D: bupropion, captopril, carbamazepine, dicyclomine, digoxin, doxepin, fentanyl, furosemide, morphine, nifedipine, olanzapine, theophylline, tiotropium bromide, and warfarin. ^a^Although they are not analgesic medications, these antispasticity medications may be used to treat pain due to uncontrolled muscle tone.

Among the 54.0% of participants with pain, 61.0% used 1 or more medication indicated for treatment of pain; 39.0% of those reported to have pain did not use any analgesic (Figure 3A). When antispasticity medications were included, 78.0% of participants used 1 or more medications indicated for the treatment of pain. Multiple analgesics were more frequently used by participants with pain compared with those without (difference, 21.0%; 95% CI, 4.0% to 38.0%) and by those with pain in worse health compared with usual health (difference, 30.0%; 95% CI, 3.0% to 57.0%). Among the 28.0% of participants with constipation, 75.0% used 1 or more medication indicated for treatment of constipation (Figure 3B). Multiple laxatives were more frequently used by participants with constipation compared with those without (difference, 15.0%; 95% CI, −4.0% to 34.0%) and by those with constipation in worse health compared with usual health (difference, 30.0%; 95% CI, −9.0% to 69.0%). For both pain and constipation, those in worse health used a more diverse set of medications.

Among the 28.0% of participants with constipation, 89.0% used 1 or more anticholinergic medications and 61.0% took 2 or more anticholinergic medications (Figure 3C). Multiple anticholinergic medications were more frequently used by participants with constipation compared with those without (difference, 7.0%; 95% CI, −14.0% to 28.0%) and by those with constipation in worse health compared with usual health (difference, 55.0%; 95% CI, 33.0% to 77.0%). Similarly, among the 35.0% of participants with drowsiness, 91.0% used 1 or more anticholinergic medication and 63.0% took 2 or more anticholinergic medications (Figure 3D). Multiple anticholinergic medications were more frequently used by participants with drowsiness compared with those without (difference, 11.0%; 95% CI, −9.0% to 31.0%) and by those with drowsiness in worse health compared with usual health (difference, 24.0%; 95% CI, −8.0% to 56.0%).

## Discussion

In this study, children with SNI experienced high symptom burdens and used a broad array of medications. The parent-reported symptoms with the highest prevalence in our study included irritability, insomnia, and pain, each of which has a recognized need for improved evidence-based treatments.^5,6,7^ The finding that children with higher symptom burdens also used increased counts and varieties of medications is consistent with analogous geriatric studies.^36^ The cross-sectional associations between specific symptoms and alleviating or exacerbating medications (eg, that only 61.0% of children with reported pain were using ≥1 pain medication) indicate that opportunities exist to support clinicians in providing personalized symptom management. Considering these findings, several issues deserve further discussion, in part to highlight the work required to advance the science of personalized symptom management in children with SNI.

First, standardized symptom assessments were made by parents who provided the bulk of direct care but were nonetheless an approximation of their child’s true experience.^14,34^ This aspect is an inherent characteristic because no reference-standard patient-reported outcomes data exist to corroborate parent assessments in children who cannot self-report.^19^ Nevertheless, the observed GSSs and their association with recent health status were consistent with other studies comparing parent and child assessments, reinforcing the face validity of symptom data.^34^ For symptoms with more objective and commonly documented physical manifestations, such as seizure or diarrhea, the reliability of symptom data could be verified by electronic health record review. For other symptoms associated with less direct physical manifestations, symptoms could be verified using available symptom-specific measurement tools, such as pain assessment tools for children who cannot communicate their experience of pain.^5^ For the most subjective remaining symptoms, such as sadness or itching, qualitative interviews may further elucidate how parents interpret and score these symptoms. Furthermore, because parents may have different reporting thresholds for the frequency, severity, and extent of bother components of the GSS, parent interviews could help establish more meaningful scaling for these measures. Regardless, parents who provide most of the direct care are in the best position to interpret and report symptoms, just as they would during usual clinical visits.

Second, our findings suggest that paired symptom-medication data could be used clinically to identify multiple potential targets for improved symptom management, including symptoms that may be underrecognized or undertreated. Despite reporting that their child was in their usual state of health, parents still reported many bothersome symptoms. Without a structured way to collect and monitor multiple symptoms and their association with reconciled medication regimens, treatable symptoms may go unaddressed during the course of a clinical visit.^37^ For example, pain was a highly prevalent reported symptom in this study, affecting more than half of all patients, and adequate treatment should be a high clinical priority.^4,5^ Of the 39.0% of those reported to have pain but not using any analgesic, this may represent an underrecognized symptom. Of the 61.0% of those reported to have pain but already taking an analgesic, this may indicate undertreatment of pain, particularly when pain is occurring during normal state of health or better. These interpretations are not meant to oversimplify the often multifactorial and complex causes of pain or to simplistically suggest that a child is receiving the wrong analgesic, needs a larger does dose, or needs an additional analgesic. Rather, these findings highlight the potential clinical advantages of systematically and reliably alerting clinicians of potential undertreated symptoms (eg, chronic pain), so that additional causes (eg, uncontrolled muscle tone) and possible treatments (eg, muscle relaxants) can be explored. The availability of paired symptom and medication data immediately before the start of a clinical visit could help clinicians refine and optimize their patients’ symptom management regimens in real time.

Finally, the substantial polypharmacy noted in our study, especially among those children with the highest symptom burdens, highlights potential consequences of polypharmacy. Almost all children in this study took neurological, gastrointestinal, and respiratory medications, which may reflect common SNI-related medical issues (eg, seizures, gastrostomy tube, or tracheostomy with ventilator dependence) that necessitate widespread use of such medications. Necessity notwithstanding, polypharmacy increases the risk of adverse effects through exposure to more potential drug-drug interactions or adverse effects, such as additive or synergistic medication effects.^12,30^ Anticholinergic symptoms, such as constipation or drowsiness, can dramatically affect a patient’s quality of life. Although a clinician may prescribe a medication with anticholinergic properties that alone would pose minimal risk, the additive effects of multiple medications with anticholinergic properties, like those commonly used in children with SNI (eg, antipsychotics, antiepileptics, or antihistamines), may be considerable.^11,27,38^ In this study, among the 28.0% of children with constipation, 61.0% were taking 2 or more anticholinergic medications, and of the 35.0% with reported drowsiness, 63.0% were taking 2 or more anticholinergic medications, raising the question whether these anticholinergic symptoms may be caused or exacerbated by medication use. Although more work needs to be done regarding the consequences of polypharmacy, this set of findings underscores the potential benefit of a system of care where symptoms would be routinely assessed and automatically cross-checked with known associations of drugs with adverse effects, with the results of this assessment reported back to clinicians and parents.

### Limitations

Our findings must be interpreted in the context of the following limitations. First, this study was conducted at a single, large, hospital-based special needs clinic and the cohort’s racial diversity differed from the general population, which may limit generalizability. This special needs clinic, however, is one of the largest in the US and has a 7-state catchment area. Furthermore, the PRO-Sx system can be scaled to support future multicenter studies among more diverse populations and clinical settings to increase generalizability. Second, the most common reasons that eligible patients did not enroll were that the medical team entered the patient room before the research assistant could broach possible enrollment or because a research assistant was unavailable. Although these implementation challenges may have resulted in selection bias, this seems unlikely. Third, our analysis relied on previsit medication lists; it is possible that parents mentioned the reported symptoms during the subsequent clinical visit, leading to optimization of postvisit medications not reflected in our results. However, concordance between parent-reported symptoms and subsequent clinician-noted symptoms was low.^37^ Fourth, by excluding as-needed medications, which can play an important role in managing symptoms such as pain, we may have underestimated the degree of pharmacotherapy a patient was receiving for a specific symptom. Fifth, the cross-sectional design of this study does not allow for assessment of causality between medications and symptoms; doing so will require longitudinal data collection and analysis.

## Conclusions

Children with SNI and polypharmacy experience high symptom burdens and use a diverse array of medications. Our cross-sectional findings associating symptoms with medications illuminate how certain symptom-medication combinations may benefit from proactive, increased monitoring. Longitudinal measurements of parent-reported symptoms should be tested in future studies as both a clinical indicator for pharmacotherapy (or other therapies and interventions) and as a clinical outcome to assess for desired or adverse therapeutic responses.

## Notes

Supplement. **eFigure 1.** Parent-Reported Outcomes of Symptoms (PRO-Sx) System **eTable.** Demographics of Parents Reporting Symptom Data for 100 Children with Severe Neurological Impairment (SNI) **eFigure 2.** Prescribed Medication Classes by Global Symptom Score (GSS) Quartiles in 100 Children with Severe Neurological Impairment (SNI)
