# Supplementary material for: Parent-Reported Symptoms and Medications Used Among Children With Severe Neurological Impairment
Source: JAMA Netw Open. 2020 Dec 11;3(12):e2029082. doi: 10.1001/jamanetworkopen.2020.29082 (PMC7733159; doi:10.1001/jamanetworkopen.2020.29082)
Supplement: Supplement. — eFigure 1. Parent-Reported Outcomes of Symptoms (PRO-Sx) System eTable. Demographics of Parents Reporting Symptom Data for 100 Children with Severe Neurological Impairment (SNI) eFigure 2. Prescribed Medication Classes by Global Symptom Score (GSS) Quartiles in 100 Children with Severe Neurological Impairment (SNI) [file jamanetwopen-e2029082-s001.pdf]

Supplemental Online Content

Feinstein JA, Feudtner C, Blackmer AB, et al. Parent-reported symptoms and medications used among children with severe neurological impairment. *JAMA Netw Open*. 2020;3(12):e2029082. doi:10.1001/jamanetworkopen.2020.29082

eFigure 1. Parent-Reported Outcomes of Symptoms (PRO-Sx) System

eTable. Demographics of Parents Reporting Symptom Data for 100 Children with Severe Neurological Impairment (SNI)

eFigure 2. Prescribed Medication Classes by Global Symptom Score (GSS) Quartiles in 100 Children with Severe Neurological Impairment (SNI)

This supplemental material has been provided by the authors to give readers additional information about their work.

© 2020 Feinstein JA et al. *JAMA Network Open.*

eFigure 1. Parent-Reported Outcomes of Symptoms (PRO-Sx) System.
This figure displays a schematic diagram of the PRO-Sx system.

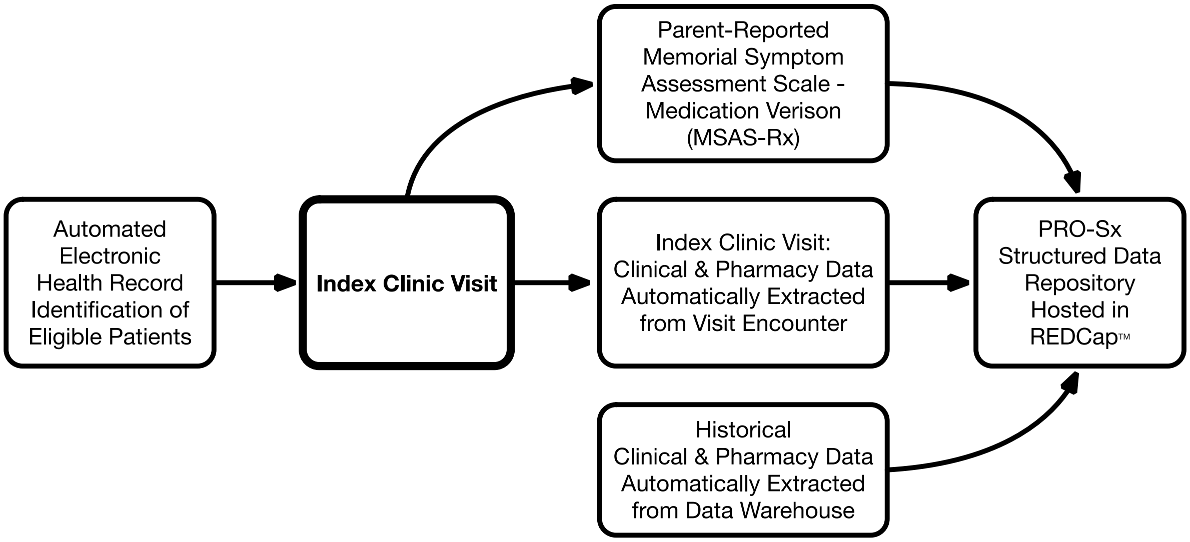

© 2020 Feinstein JA et al. *JAMA Network Open.*

eTable. Demographics of Parents Reporting Symptom Data for 100 Children with Severe Neurological Impairment (SNI)

	**Overall (%)** N=100
**Parent Age (years)**	
18-30	17.0
31-40	47.0
41-50	27.0
>50	8.0
Not Specified	1.0
**Sex**	
Male	16.0
Female	83.0
Not Specified	1.0
**Race**	
American Indian/Alaska Native	1.0
Asian	1.0
Black/African American	4.0
White	78.0
More than one race	6.0
Not Specified	10.0
**Ethnicity**	
Not Hispanic or Latino	75.0
Hispanic or Latino	23.0
Not Specified	2.0
**Parent Language**	
English	95.0
Spanish	2.0
Other	3.0
**Parent Education**	
Some High School	5.0
High School Graduate	15.0
Some College/Technical School	39.0
College Graduate	40.0
Not Specified	1.0
**Parent Annual Income**	
<$15,000	10.0
$15,000 to <$35,000	17.0
$35,000 to <$50,000	7.0
$50,000 to <$75,000	19.0
>$75,000	34.0
Not Specified	13.0
**Parent-Provided Care (Days Per Week)**	
0	0.0
1-3	4.0
4-6	4.0
7	89.0
Not Specified	3.0
**Nurse-Provided Care (Days Per Week)**	
0	48.0
1-3	17.0
4-6	18.0
7	14.0
Not Specified	3.0

© 2020 Feinstein JA et al. *JAMA Network Open.*

eFigure 2. Prescribed Medication Classes by Global Symptom Score (GSS) Quartiles in 100 Children with Severe Neurological Impairment (SNI).

The percentage displayed in each heatmap square corresponds to the percentage of children within each increasing GSS score quartile (Y-axis) who utilized ≥1 medication from an Anatomic Therapeutic Chemical (ATC) medication class (X-axis, ordered by descending overall medication utilization).

	Alimentary Tract Agents	Respiratory Agents	Nervous System Agents	Musculoskeletal Agents	Blood & Blood Forming Organs	Cardiovascular Agents	Systemic Hormones	Genitourinary & Sex Hormones	Systemic Anti-Infectives	Antineoplastic Agents	Antiparasitic Agents
GSS- 1st Quartile (N=26)	92	85	77	23	31	38	23	35	19	0	0
GSS- 2nd Quartile (N=24)	96	88	88	54	33	29	17	25	21	8	0
GSS- 3rd Quartile (N=25)	96	96	84	44	36	32	40	12	36	0	0
GSS- 4th Quartile (N=25)	100	80	96	48	32	28	36	28	24	0	4

© 2020 Feinstein JA et al. *JAMA Network Open.*
